# Randomized study of two endo-knives for the traction-assisted endoscopic submucosal dissection of early esophageal squamous cell carcinoma

**DOI:** 10.1038/s41598-022-08348-0

**Published:** 2022-03-17

**Authors:** Yoshiyasu Kitagawa, Asuka Ishigaki, Rino Nishii, Osamu Sugita, Takuto Suzuki

**Affiliations:** Endoscopy Division, Chiba Cancer Center, 666-2 Nitonacho, Chuo-ku, Chiba, Japan

**Keywords:** Oesophageal cancer, Oesophageal cancer

## Abstract

Needle-type devices, such as the DualKnife (Olympus, Tokyo, Japan), are widely used for traction-assisted esophageal endoscopic submucosal dissection (ESD) but require a prolonged operation time. An improved model of the ITknife (Olympus), the ITknife nano, may allow faster and easier ESD than the DualKnife. We conducted a randomized study to compare the performances of the DualKnife and the ITknife nano for traction-assisted esophageal ESD. Patients with early esophageal squamous cell carcinoma were eligible for this study. The primary outcome was the total procedure time. The secondary outcomes were submucosal dissection time, en bloc, and complete resection rates, perforation rate, and adverse events. Results Fifty patients were equally divided into two groups: the DualKnife group (D-group) and the ITknife nano group (I-group), and all underwent the assigned treatment. The I-group had significantly shorter total procedure time (36.8 vs. 60.7 min; *P* < 0.01) and submucosal dissection time (17.2 vs. 35.8 min; *P* < 0.01) than the D-group. The en bloc and complete resection rates were sufficiently high in both groups (100% and 100% in the D-group and 100% and 96% in the I-group, respectively). Significantly fewer hemostatic procedures due to intraoperative bleeding were performed in the I-group than in the D-group (0.2 vs. 1.4; *P* < 0.01). Delayed bleeding, perforation, or esophageal stricture did not occur in either group. The ITknife nano exhibited lower procedure time for traction-assisted esophageal ESD than the DualKnife, without increasing adverse events.

## Introduction

Endoscopic resection is an accepted and established treatment procedure for early-stage esophageal cancer with a negligible risk of lymph node metastasis^1^. Endoscopic submucosal dissection (ESD) shows considerable advantages regarding en bloc resection rate, curative resection rate, and local recurrence compared with endoscopic mucosal resection (EMR)^2,3^. However, it is not universally applied because it is technically more demanding and time-consuming (compared with EMR) and is associated with a higher complication rate, especially perforation^4,5^. In particular, esophageal ESD is considered more technically challenging than gastric ESD because of difficulties associated with anatomical features of the esophagus.

To reduce the risk of adverse events related to ESD, researchers developed various traction methods^6,7^. The clip-with-line traction method (which uses a hemoclip with a long thread) is one of the most popular methods, and its feasibility has been previously reported^8–13^. Needle-type devices such as the DualKnife (Olympus, Tokyo, Japan) have demonstrated favorable results for traction-assisted esophageal ESD^2,3,5,10,11^. However, traction-assisted esophageal ESD has not been sufficiently standardized. It can still lead to injury of the muscularis propria, and requires a prolonged operation time^10,11^.

An improved model of the ITknife (Olympus), the ITknife nano, has recently become available. This can be used for both incisions and dissections, and is well suited for lesions in the esophagus and colon^14^. The device has a long arm, which allows rapid dissection, and an insulated tip to avoid inadvertent injury to the muscular layer. We have previously reported the safety and efficacy of using the ITknife nano in traction-assisted esophageal ESD^15^. It is quite different than the DualKnife, and may be able to realize ESD faster and more easily.

No randomized-controlled trial has yet investigated whether the ITknife nano has improved technical outcomes in traction-assisted esophageal ESD, compared with those of the DualKnife. Therefore, we conducted prospective, randomized clinical trial to compare their performances in traction-assisted esophageal ESD, in terms of speed and safety.

## Methods

### Study design and participants

This randomized-controlled trial was conducted at the Chiba Cancer Center (Chiba, Japan) in accordance with the ethical principles of the Declaration of Helsinki (Fortaleza revision) and in compliance with the ethical guidelines for medical and health research involving human subjects in Japan. This study was begun after approval by the Ethics Committee of the Chiba Cancer Center and registration in the University Hospital Medical Network Clinical Trial Registry (No. UMIN000033156) on 27/06/2018. Written informed consent was obtained from all patients before enrollment.

Patients who met the eligibility criteria were enrolled from July 2018 to July 2020. The inclusion criteria were: (1) histologically diagnosed or suspected squamous cell carcinoma, according to biopsy specimens; (2) lesion > 11 mm in size; (3) clinically diagnosed intramucosal cancer; (4) no lymph node or distant metastasis at preoperative evaluation with computed tomography (CT); (5) patient aged ≥ 20 years; (6) Eastern Cooperative Oncology Group performance status ≤ 1; (7) no prior history of esophagectomy for esophageal cancer; (8) no prior treatment with chemotherapy or radiation. The exclusion criteria were as follows: (1) Active infection requiring systemic therapy; (2) severe organ failure; (3) history of iodine hypersensitivity; (4) lesions ≤ 10 mm from the scar after endoscopic resection.

### Randomization

The participants were randomly assigned to the D-group or I-group at a 1:1 allocation ratio. Randomization was performed by using web-based software (Mujinwari; Iruka System, Tokyo, Japan). Further randomization was applied for the stratification of tumor location (upper, middle, and lower third), tumor size (< 30 and ≥ 30 mm diameter), esophageal circumference (< 1/2 and ≥ 1/2), and endoscopist. Participants and endoscopists were not blind to the allocated group.

### ESD procedure

The procedures were conducted by three board-certified endoscopists who had experience in at least 50 esophageal, 200 gastric, and 100 colorectal ESD procedures. In our hospital, the DualKnife is mainly used for colorectal ESD, whereas the ITknife2 is mainly used for gastric ESD. For esophageal ESD, DualKnife or ITknife nano was used at discretion of the endoscopist before starting the study. The endoscopists had sufficient experience in esophageal ESD using both devices. All patients were treated under conscious sedation with midazolam and pethidine. If conscious sedation was considered inappropriate before starting ESD, because of the patient’s condition or expected long duration of the procedure (> 90 min), general anesthesia was used. An endoscope with water-jet function (GIF-Q260J or GIF-H290T; Olympus) was used with an attachment (D-201-11804; Olympus). Carbon dioxide insufflation was used. Lugol chromoendoscopy was performed before marking the lateral margin of the lesion. Marking dots were placed 2–3 mm outside of the margins of the lesion using electrocoagulation. Next, hyaluronic acid solution (MucoUp; Johnson & Johnson, Tokyo, Japan) was injected into the submucosal layer. In the D-group, a mucosal incision of the anal side was made using a Dual Knife, and circumferential incisions were made using a Dual Knife in endocut I mode (effect, 2; duration, 2; interval, 2) using a VIO 300D (Erbe, Tubingen, Germany). In the I-group, a mucosal incision was made using a free needle knife (KD-1L-1; Olympus), and circumferential incisions were made using an ITknife nano with endocut Q mode (effect, 3; duration, 2; interval, 1).

After the circumferential incisions and trimming the submucosa at the edge of the incision, the endoscope was withdrawn. Thereafter, preparations were made for the clip traction method. A ZEOCLIP^™^ delivery catheter (ZP-S-165S; Zeon Medical, Tokyo, Japan) was inserted into the accessory channel of the endoscope, and a ZEOCLIP (ZP-CH; Zeon Medical) was mounted onto the tip of the delivery catheter. A long nylon thread was passed through the side hole of the clip and it was withdrawn into the endoscope. After insertion, the clip was anchored to the oral edge of the specimen. During submucosal dissection, the traction thread was weighted by a sinker of approximately 10 g.

**Figure 1:**
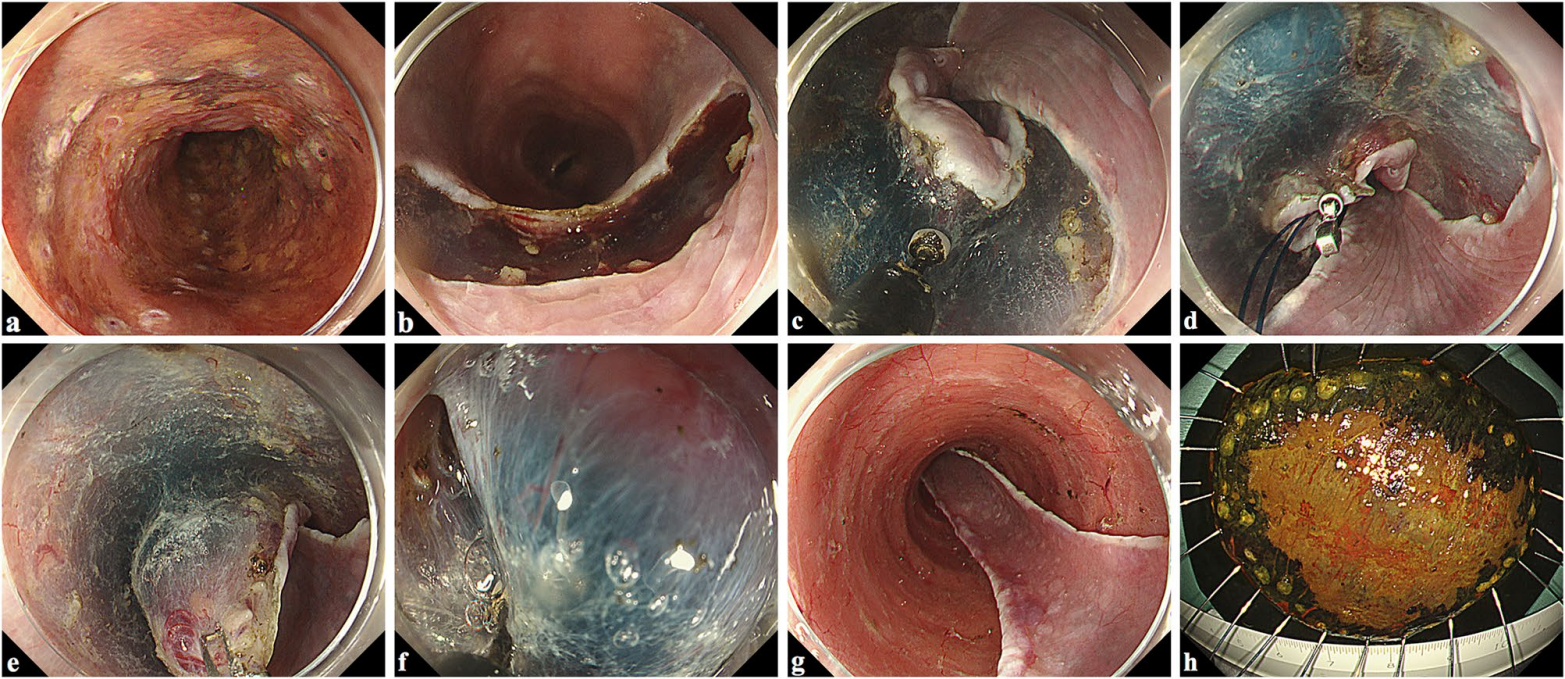
Steps of traction-assisted endoscopic submucosal dissection for large esophageal cancer. (**a**) Marking dots were made after lugol chromoendoscopy. (**b**) The anal incision was performed. (**c**) The circumferential incisions and trimming the submucosa at the edge of the incision were completed. (**d**) The traction clip was anchored to anchored to the oral edge of the specimen. (**e**) During submucosal dissection, the traction thread was pulled in the oral direction with a sinker. (**f**) The clip-with-line traction method provides good visualization and tension of the submucosa. (**g**) After resection of the tumor. (**h**) Resected specimen after iodine staining. Complete en bloc resection is achieved.

In the D-group, submucosal dissection was completed using a DualKnife in swift coagulation mode (effect, 3; 30 W). In the I-group, submucosal dissection was completed using an ITknife nano with a swift coagulation mode (effect, 5; 45 W). To control bleeding, a Coagrasper (FD-410LR; Olympus) was used in soft coagulation mode (effect, 5; 50 W). Locoregional steroid injection of triamcinolone acetonide was administered for patients with a mucosal defect encompassing over two-thirds of the esophageal circumference. Triamcinolone acetonide (Kenacort; Bristol-Myers Squibb, Tokyo, Japan) was diluted with saline, and 40–80 mg was injected into the residual submucosa just after ESD, according to the method proposed by Hanaoka et al.^16^. For lesions of the entire circumference, steroid injection combined with oral prednisolone was administered^17^. Oral intake of prednisolone was started at 30 mg/day and tapered gradually for weeks according to the method of Yamaguchi et al.^18^. Figure 1 and Video 1 show the steps of the traction-assisted esophageal ESD.

### Study end points

The primary outcome variable was total procedure time. The secondary outcome variables were submucosal dissection time, rate of en bloc resection, rate of complete resection, number of hemostatic therapy events using the Coagrasper, and adverse events (muscular layer exposure, perforation, delayed bleeding, and esophageal stricture).

Total procedure time was defined as the time from the start of submucosal injection to the end of tumor removal. Submucosal dissection time was defined as the time of anchoring the clip to the end of tumor removal. Resections were defined as en bloc when the lesion was resected in one piece and the resection margins were macroscopically tumor-free. Complete resection was defined as removal of the entire tumor in one piece with no histopathologic evidence of the tumor at the resection margins. Hemostasis events were counted only when the Coagrasper was required for hemostasis of intraoperative bleeding. Muscle layer exposure was defined as when the fine texture of the muscle fibers of muscularis propria was clearly exposed and visible endoscopically, due to deep submucosal dissection. Esophageal perforation was defined as a visible hole in the esophageal wall that exposed the mediastinal cavity. Delayed bleeding was defined as clinical evidence of bleeding after ESD, requiring blood transfusion or endoscopic or surgical intervention. Esophageal stricture was defined as when a standard endoscope for upper gastrointestine, 9.9 mm in diameter (GIF-Q260J; Olympus), could not be passed through the treatment site. After ESD, all patients underwent follow-up endoscopic examinations at least every 6 months.

Subgroup analysis was performed to compare the therapeutic outcomes between small and large lesions. Small lesions were defined as tumor size < 30 mm in diameter and esophageal circumference < 1/2. Large lesions were defined as tumor size ≥ 30 mm in diameter or esophageal circumference ≥ 1/2. Subgroup analysis was also performed to compare total procedure times among subgroups, divided according to location of lesion, endoscopist, sedation, and study period (earlier 25 cases; later 25 cases).

### Statistical analysis

The sample size was calculated based on the initial results for esophageal ESD in our center. Our mean procedure times for the traction-assisted esophageal ESD were 60 min using the DualKnife (standard deviation, 24). We estimated that the mean procedure times using the ITknife nano would be 40 min, based on previous report. A total of 24 lesions were required for each group to detect a significant difference between the groups, based on a two-sided test with a significance level of 0.05 and a power of 80%. We estimated that 50 lesions would be required, considering a 5% dropout.

The results of this study are expressed as mean ± standard deviation. Continuous variables were compared between-group differences using the Welch t-test. Categorical variables were compared between treatment groups using chi-square or Fisher’s exact test, as appropriate. Differences were considered significant at a *P* value of < 0.05. All analyses were performed using SPSS version 11.0 J (SPSS, Chicago, IL, USA).

## Results

### Patient characteristics

**Figure 2:**
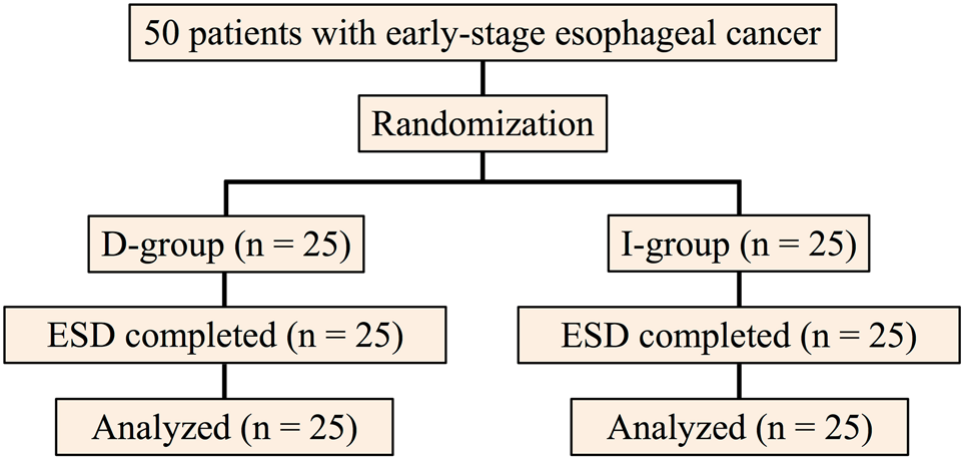
Study flowchart showing patient inclusion and randomization. *D-group* DualKnife group, *I-group* ITknife nano group, *ESD* endoscopic submucosal dissection.

50 patients with early-stage esophageal cancer were enrolled, of whom 25 and 25 patients were assigned to the D-group and I-group, respectively. All patients underwent ESD. Fifty patients were included in the analysis (Fig. 2).

The baseline characteristics were well balanced between the two groups with respect to age, gender, tumor size, tumor location, macroscopic type, esophageal circumference, histologic depth of tumor, endoscopist, and sedation (Table 1).

**Table 1:** Baseline characteristics of between the D-group and the I-group.

	D-group (n = 25)	I-group (n = 25)	*P* value
Age, mean (± SD), years	71.2 (± 8.6)	72.7 (± 6.2)	0.49
Sex, male/female	22 (88)/3 (12)	21 (84)/4 (16)	1.00
**Estimated tumor size**			0.75
< 30, mm	17 (68)	19 (76)	
≥ 30, mm	8 (32)	6 (24)	
**Location of lesions**			0.37
Upper third	5 (20)	3 (12)	
Middle third	16 (64)	14 (56)	
Lower third	4 (16)	8 (32)	
**Macroscopic type**			0.50
II c	15 (60)	12 (48)	
II b	9 (36)	10 (40)	
II a	1 (4)	3 (12)	
**Circumference of the lesion**			1.00
< 1/2	20 (80)	21 (84)	
≥ 1/2	5 (20)	4 (16)	
**Histologic depth of tumor**			0.36
EP/LPM	23 (92)	22 (88)	
MM/SM1	1 (4)	3 (12)	
SM2	1 (4)	0 (0)	
**Endoscopists**			0.68
A	16 (64)	15 (60)	
B	7 (28)	6 (24)	
C	2 (8)	4 (16)	
**Sedation**			0.43
Conscious sedation	21 (84)	23 (92)	
General anesthesia	4 (16)	2 (8)	

Values are n (%) unless otherwise indicated.

*D-group* DualKnife group, *I-group* ITknife nano group, *SD* standard deviation, *EP* carcinoma in situ, *LPM* tumor invasion into the lamina propria mucosa, *MM* tumor invasion into the muscularis mucosa, *SM1* tumor invasion into the upper third of the submucosal layer, *SM2* tumor invasion into the middle third of the submucosal layer.

### Study outcomes

The ESD procedure was completed in both groups.

Table 2 shows the procedure-related outcomes between the D-group and the I-group. The total procedure time in the I-group was significantly shorter than in the D-group (36.8 ± 20.8 vs. 60.1 ± 40.6 min, respectively; *P* < 0.01). Submucosal dissection time in the I-group was significantly shorter than in the D-group (17.2 ± 13.9 vs. 35.8 ± 30.9 min, respectively; *P* < 0.01).

**Table 2:** Comparison of procedure-related outcomes between the D-group and the I-group.

	D-group (n = 25)	I-group (n = 25)	*P* value
**Total procedure time**			
Mean (± SD), min	60.1 (± 40.6)	36.8 (± 20.8)	0.01
Median (range), min	48 (21–167)	31 (11–88)	0.01
**Submucosal dissection time**			
Mean (± SD), min	35.8 (± 30.9)	17.2 (± 13.9)	0.01
Median (range), min	27 (27–118)	11 (4–54)	0.01
Specimen size, mean (± SD), mm	40.5 (± 14.0)	38.0 (± 8.1)	0.46
En bloc resection	25 (100)	25 (100)	1.00
Horizontal margin involvement	0 (0)	1 (4)	1.00
Vertical margin involvement	0 (0)	0 (0)	1.00
Complete resection	25 (100)	24 (96)	1.00
**Number of hemostatic therapy**			
Mean (± SD)	1.4 (± 1.9)	0.2 (± 0.5)	< 0.01
Median (range)	1 (0–6)	0 (0–2)	< 0.01
**Adverse events**			
Delayed bleeding	0 (0)	0 (0)	1.00
Muscular layer exposure	5 (20)	2 (8)	0.26
Perforation	0 (0)	0 (0)	1.00
Stricture	0 (0)	0 (0)	1.00

Values are n (%) unless otherwise indicated.

*D-group* Dual knife group, *I-group* IT knife nano group, *SD* standard deviation.

There were no significant differences between the two groups with respect to specimen size. En bloc resection rates were 100% in both groups. One horizontal margin involvement was observed in the I-group, although en bloc resection was achieved without inadvertent intralesional incision. Histopathological diagnosis revealed that esophageal cancer extended beyond the ESD marking dots. Follow-up endoscopy every 3 months was cautiously performed on lesions with a positive horizontal margin, and no local recurrence was detected. No vertical margin involvement was observed in either group. Complete resection rates were sufficiently high in both groups (100% in the D-group and 96.0% in the I-group). Significantly fewer hemostatic procedures caused by intraoperative bleeding were performed in the I-group than in the D-group (0.2 ± 0.5 vs. 1.4 ± 1.9, respectively; *P* < 0.01).

No delayed bleeding occurred in either group. Slightly fewer muscular layer exposures were observed in the I-group, compared with the D-group, but the difference was insignificant (20% vs. 8%, respectively; *P* = 0.42). Perforation or esophageal stricture did not occur in either group.

### Subgroup analysis

Table 3 shows the results of subgroup analyses of total procedure time in terms of various clinical characteristics.

**Table 3:** Subgroup analyses for total procedure times in terms of various clinical characteristics.

	D-group	I-group	*P* value
**Lesion size**			
Small lesion	38.0 (± 14.4)	27.4 (± 11.0)	0.02
Large lesion	108.9 (± 36.0)	66.5 (± 15.7)	0.02
**Location of lesions**			
Upper third	45.0 (± 19.1)	35.7 (± 12.9)	0.51
Middle third	64.4 (± 39.7)	34.8 (± 21.8)	0.02
Lower third	65.5 (± 56.2)	40.6 (± 20.7)	0.51
**Endoscopists**			
A	70.3 (± 45.1)	42.7 (± 21.8)	0.04
B	39.6 (± 23.2)	21.0 (± 7.8)	0.11
C	58.0 (± 11.0)	38.3 (± 17.6)	0.29
**Sedation**			
Conscious sedation	52.4 (± 33.3)	36.4 (± 20.9)	0.07
General anesthesia	104.0 (± 47.5)	40.5 (± 18.5)	0.13
**Study period**			
Early	72.0 (± 40.2)	44.3 (± 22.9)	0.05
Later	48.4 (± 37.4)	29.8 (± 15.6)	0.15

Values are mean (± standard deviation).

*D-group* DualKnife group, *I-group* ITknife nano group.

With respect to small lesions, the total procedure time in the I-group was significantly shorter than in the D-group (27.4 ± 11.0 vs. 38.0 ± 14.4 min; *P* = 0.02). With respect to large lesions, the total procedure time in the I-group was significantly shorter than in the D-group (66.5 ± 15.7 vs. 108.9 ± 36.0 min; *P* = 0.02).

Total procedure times in the I-group were shorter than those in the D-group for all characteristics, including location of lesion, endoscopist, sedation, and study period. While some differences were not statistically significant, the consistency of the results was confirmed by subgroup analyses.

## Discussion

The purpose of our study was to investigate whether the ITknife nano improved procedure-related outcomes of traction-assisted esophageal ESD, compared with the DualKnife. We demonstrated that ITknife reduced the total procedure time without increasing the risk of adverse events.

A variety of traction methods are available to facilitate a faster, safer procedure during ESD^6,7^. The clip-with-line method allows for adequate proximal traction in esophageal ESD, allowing good visualization and tension of the submucosa^8–13^. Several studies have reported the effectiveness of traction-assisted ESD in the esophagus^10,11,13^. In a single-center, prospective, randomized study, Koike et al. reported that significant shortening of dissection time was achieved in traction-assisted ESD, compared with conventional ESD^11^. In a multicenter, prospective, randomized study, Yoshida et al. demonstrated that traction-assisted ESD was superior to conventional ESD in terms of total procedure time, for large lesions^13^. In light of these findings, it is strongly recommended to use the clip-with-line traction method for esophageal ESD.

With regard to endoscopic knives for esophageal ESD, a single-arm study reported the high performances of needle-type devices, such as the HookKnife™ (Olympus), Flush Knife (Fuji, Tokyo, Japan), and DualKnife, with favorable en bloc resection rates of 95%–100% and perforation rates of 0–6.9%^2,3,19,20^. We previously reported the effectiveness and safety of the ITknife nano for esophageal ESD, with rates of en bloc resection and perforation of 100% and 0%, respectively^15^. The performances of different endoscopic knives for esophageal ESD have previously been compared only against conventional ESD, in which the Mucosectom knife (PENTAX, Tokyo, Japan) reduced the total procedure time and the submucosal dissection time, with lower complication rates, compared with the FlushKnife^21^. Therefore, we conducted a randomized prospective trial to compare the performance of two endoscopic knives for traction-assisted esophageal ESD.

We selected the DualKnife and the ITknife nano from among the various endoscopic knives available. The DualKnife is a needle-type device, mainly used in our hospital for colorectal ESD^14^. The ITknife nano is a refined version of the ITknife2, which is most commonly used for gastric ESD^22^; because of the smaller insulation tip and disk blade, submucosal dissection in the forward direction under direct observation is possible in the narrow lumen of the esophagus. In this study, all procedures were conducted by board-certified endoscopists who had sufficient experience of gastric and colorectal ESD; this ensured quality and minimized any learning curve effects.

The total procedure times were 40% shorter for the I-group than for the D-group, with no adverse events in the I-group. In subgroup analysis of endoscopist and study period, some differences were not statistically significant, but this is considered to be due to the small number of cases. Consistency in the result of shorter procedure times in the I-group was confirmed. Owing to the ITknife nano’s design features, procedures seemed technically easy and safe in three respects. First, the ITknife nano uses a long arm extending from the disk, which allows the operator to dissect the tissue widely. Second, the long arm also provides effective hemostasis, which contributes to reducing intraoperative bleeding—significantly fewer hemostatic procedures due to intraoperative bleeding were performed in the I-group than in the D-group—thereby avoiding the need to change endoscopic devices. Third, an insulated tip prevents inadvertent electrical contact between the blade and the deeper layers of the esophageal wall. Also, the metal disk at the back of an insulated tip can hold and stabilize the device while maintaining tension on the submucosa. Using needle-type devices, it took more time to dissect the submucosal fibers in stages, while taking care not to injure the muscle layer. Although the difference is not statistically significant, we observed a tendency toward decreased muscular layer exposure using the ITknife nano.

This study has several limitations. First, we could not blind the endoscopists to treatment group allocation, which might have led to performance bias. However, total procedure time in the DualKnife group was similar to outcomes previously reported for other needle-type devices^2,3^. Second, the current study did not use the DualKnife with a water-jet function. The performances of different endoscopic knives for esophageal ESD have previously been compared in an animal model in which the needle-type device with a water-jet function was safer (with less bleeding and perforation) than a conventional knife^23^. However, a comparative study in a clinical setting demonstrated that a blade-type device was superior to a needle-type device with a water-jet function, in terms of procedure duration for esophageal ESD, without increasing adverse events^21^. Moreover, traction-assisted ESD requires a smaller volume of injection fluid, because vertical traction helps maintain adequate endoscopic visualization of the submucosal layer. Therefore, benefit from a water-jet function would be limited in traction-assisted esophageal ESD. Further study should clarify assess the effectiveness of ITknife nano compared with endoscopic knives with water-jet function. Third, this study was limited by the use of three expert endoscopists in a single center. A multicenter trial including novice operators may be required to standardize traction-assisted ESD globally. Finally, because we excluded lesions with prior treatment for esophageal cancer, the effectiveness of the ITknife nano remains to be clarified for traction-assisted esophageal ESD for lesions with submucosal fibrosis.

This randomized-controlled trial is the first to compare the performances of two different types of endoscopic knives for traction-assisted esophageal ESD. The ITknife nano exhibited lower procedure time for traction-assisted esophageal ESD than the DualKnife, without increasing adverse events. The ITknife nano with the clip-with-line traction method may serve as a part of standard procedure for esophageal ESD.

## Supplementary Information

Supplementary Video 1.
